# *Syn3* Gene Knockout Negatively Impacts Aspects of Reversal Learning Performance

**DOI:** 10.1523/ENEURO.0251-21.2021

**Published:** 2021-09-08

**Authors:** Alyssa Moore, Jérôme Linden, James D. Jentsch

**Affiliations:** 1 Department of Psychology, Binghamton University, Binghamton, New York 13902; 2 Department of Psychiatry and Biobehavioral Sciences, David Geffen School of Medicine, University of California, Los Angeles, Los Angeles, California 90095

**Keywords:** *Syn3*, dopamine, reversal learning, behavioral flexibility, impulsivity

## Abstract

Behavioral flexibility enables the ability to adaptively respond to changes in contingency requirements to maintain access to desired outcomes, and deficits in behavioral flexibility have been documented in many psychiatric disorders. Previous research has shown a correlation between behavioral flexibility measured in a reversal learning test and *Syn3*, the gene encoding synapsin III, which negatively regulates phasic dopamine release. *Syn3* expression in the hippocampus, striatum, and neocortex is reported to be negatively correlated with reversal learning performance, so here, we used a global knock-out line to investigate reversal learning in mice homozygous wild type, heterozygous null, and homozygous null for the *Syn3* gene. Compared with wild-type animals, we found a reversal-specific effect of genetic *Syn3* deficiency that resulted in a greater proportional increase in trials required to reach a preset performance criterion during contingency reversal, despite no observed genotype effects on the ability to acquire the initial discrimination. Behavioral flexibility scores, which quantified the likelihood of switching subsequent choice behavior following positive or negative feedback, became significantly more negative in reversal only for *Syn3* homozygous-null mice, suggesting a substantial increase in perseverative behavior in the reversal phase. *Syn3* ablation reduced the number of anticipatory responses made per trial, often interpreted as a measure of waiting impulsivity. Overall, *Syn3* expression negatively affected behavioral flexibility in a reversal-specific manner but may have reduced waiting impulsivity.

## Significance Statement

Adaptations to changes in the environment are facilitated by behavioral flexibility, and inflexible behavior is observed in several mental health disorders. The *Syn3* gene encodes synapsin III, a protein that negatively regulates phasic dopamine release by sequestering vesicles away from the ready-releasable pool. Previous research has shown a positive genetic correlation between *Syn3* expression in brain and behavioral flexibility in a reversal learning task. Here, we show that mice carrying null alleles of the *Syn3* gene exhibit less flexible responding following contingency reversal. These data reveal novel information about genetic mechanisms that may contribute to the impaired flexibility observed in multiple psychiatric conditions.

## Introduction

Behavioral flexibility relates to an individual’s ability to modify behavioral patterns in changing environmental conditions. Deficits in behavioral flexibility have been characterized in several psychiatric disorders (Uddin, 2021), including schizophrenia (Waltz and Gold, 2007; Waltz, 2017), autism spectrum disorder (Kelly and Reed, 2021), obsessive-compulsive disorder (Gruner and Pittenger, 2017; Vaghi et al., 2017), and substance use disorders (Ersche et al., 2010; Winstanley et al., 2010; Robbins et al., 2012; Istin et al., 2017).

Reversal learning is an operant test of behavioral flexibility in which an initial association (stimulus–response or response–outcome) is learned through reinforcement, before the conditions for reinforcement are reversed and the organism is tested for its ability to update behavior (Izquierdo and Jentsch, 2012; Izquierdo et al., 2017). In other words, one response is established as prepotent during initial acquisition through positive feedback and must then be inhibited or changed during reversal testing to procure reward.

Laughlin et al. (2011) evaluated reversal learning in a panel of BXD mouse strains and used a genome-wide linkage approach to model the impact of genetic variation on the reversal phenotype. A genome-wide quantitative trait locus (QTL) on mouse chromosome 10 was identified, and *Syn3*, the gene encoding synapsin III, emerged as a positional candidate expressed from that genomic region. *Syn3* mRNA expression is regulated in *cis*, and its expression in the hippocampus, neocortex, and striatum was found to be positively genetically correlated with reversal learning performance in the BXD panel, such that greater *Syn3* expression associated with faster reversal learning (Laughlin et al., 2011). Synapsin III is a member of the synapsin family of neuronal phosphoproteins (Kao et al., 1998). Synapsin III can be localized on the cytoplasmic side of synaptic vesicles and is implicated in neurotransmitter release. Feng et al. (2002) demonstrated that loss of synapsin III led to larger vesicular recycling pools but did not alter vesicular release or quantal dynamics. Synapsin proteins regulate a distal reserve pool of vesicles (Greengard et al., 1993; Hilfiker et al., 1999, 2005). Functionally, a loss of synapsin III prevents vesicles from being sequestered away from the ready-releasable pool, promoting more sustained release during continued stimulation. In a typical case, sustained release is limited by the rate of transfer of vesicles from the reserve pool to the active zone; this rate-limiting process is theoretically disrupted in cells lacking synapsin III as vesicles are inadequately sequestered in the reserve.

Synapsins are differentially expressed in neuronal populations. Bogen et al. (2006) found that selective deletion of synapsin I and II substantially reduced vesicular uptake of GABA and glutamate but did not alter dopamine (DA) uptake. Deletion reduced the concentration of vesicular transporters related to glutamate and GABA, but not vesicular monoamine transporter 2, the transporter responsible for packaging dopamine. Further, synapsin I and II colocalized in cells expressing vesicular transporters for GABA and glutamate, but not in dopaminergic terminals. A subsequent study used a triple knock-out approach to investigate differential regulation of dopamine and serotonin by synapsins (Kile et al., 2010): serotonin was not altered by the deletion of all three synapsins, but DA release was significantly enhanced. Selective deletion of synapsin III also elicited enhanced DA release, demonstrating a distinct role for the synapsins in regulating neurotransmitter release. Because DA is extensively implicated in neuropsychiatric disorders, subcellular proteins, which contribute to dopamine dynamics, and the genes encoding them are of interest.

The action of dopamine on its cognate receptors in corticostriatal systems are functionally implicated in behavioral flexibility and reversal learning. Inactivation of the D_1_-mediated direct pathway of the basal ganglia interfered with the acquisition of novel and reversed contingencies, while inactivation of the D_2_-mediated indirect pathway interfered selectively with reversal performance by increasing perseverative errors (Yawata et al., 2012). Selective deletion of presynaptic D_2_ receptors also tended to impair reversal performance and increased the number of attempts required to complete a sustained observing response (OR) to initiate a trial (Linden et al., 2018), a deficit related to waiting impulsivity (Dalley and Ersche, 2019). Groman et al. (2011) found that D_2_ receptor availability in the caudate and putamen of vervet monkeys was correlated with reversal learning performance and sensitivity to positive feedback. In a study examining DA in a compulsivity-relevant behavioral phenotype, Barker et al. (2013) found that inhibiting D_1_ or activating D_2_ in the infralimbic cortex promotes behavioral flexibility. D_2_ agonism in the nucleus accumbens impairs flexibility (Haluk and Floresco, 2009), as does systemic antagonism of D_2_/D_3_ receptors (Lee et al., 2007). Human reversal learning performance was also found to correlate with the activation of an OFC–amygdala pathway mediated by D_2_ receptors (van der Schaaf et al., 2013). These data indicate that there are region- and task-specific implications for DA in behavioral flexibility.

In this study, we investigated the role of *Syn3* on reversal learning performance using a genetic knock-out strategy. Mice expressing 0, 1, or 2 functional *Syn3* alleles were tested for reversal learning. We hypothesized that *Syn3* function would be related to behavioral flexibility; mice lacking functional *Syn3* would exhibit deficits in reversal learning performance without showing impairments in the acquisition of the initial discrimination.

## Materials and Methods

### Animals

C57BL/6N-Syn3^tm1.1(KOMP)Vlcg^/J mice (RRID:MMRRC_049950-UCD) were obtained from The Jackson Laboratory. This strain was developed by the Knockout Mouse Project (KOMP) and harbors a reporter-tagged deletion allele. This strain has been phenotyped by KOMP, with the results of those studies being publicly available at the International Mouse Phenotyping Consortium website (https://www.mousephenotype.org/data/genes/MGI:1351334#phenotypesTab).

Mice in the present study were maintained through heterozygote crosses, producing all three genotypes (homozygous null, heterozygous, or homozygous wild type, having 0, 1, or 2 functional *Syn3* alleles, respectively) in each litter. They were housed in a temperature- and humidity-controlled vivarium on a 12 h light/dark cycle, with all procedures being conducted in the light phase. Mice were weaned on postnatal day 21, at which point they were housed in same-sex groups of three to five mice per cage. Offspring were genotyped for dosage of wild-type and mutant *Syn3* alleles by Transnetyx from ear tissue collected at weaning.

A total of 112 mice offspring were involved in the study, but 6 were removed because they failed to meet preset discrimination performance criteria or because of experimenter errors. Mice that did not acquire the initial discrimination within 30 sessions were excluded from analysis (*n* = 2, both heterozygous null). Four mice were excluded because of experimenter error. The final number of mice included in the forthcoming analyses was 106: 54 females (26 homozygous null, 14 heterozygous null, and 14 homozygous wild type), and 52 males (11 null, 28 heterozygous, and 13 wild type). All protocols were reviewed and approved by the relevant Institutional Animal Care and Use Committee, and all procedures were carried out consistent with the *Guide for the Care and Use of Laboratory Animals* (National Research Council, 2011).

### Reversal learning

The parameters of the reversal task were selected to match those reported in the study by Laughlin et al. (2011), which identified *Syn3* as a candidate gene. Before the onset of these studies, mice were briefly handled daily to be weighed and tail marked. Subsequently, mice were food restricted to ∼85% of their free feeding body weights; mice were fed once per day, after testing, an amount that was individually titrated to achieve this reduced weight. Before the start of the experiment, mice were offered ∼0.5 g of reinforcer pellets (14 mg of Dustless Precision pellets; stock #F05684, Bio-Serv) per mouse in their home cage to familiarize them with the reward before operant exposure. All mice of all genotypes consumed the reward pellets in their home cages.

All testing took place in operant conditioning chambers (model MED-NP5M-D1, Med Associates), each enclosed in a sound-attenuating cubicle. The chambers were equipped with a house light and white noise generator, located outside of the chamber but within the cubicle. A photocell-equipped food delivery magazine connected to a pellet dispenser were on one wall of the chamber, and a horizontal array of five nose-poke apertures were on the opposite wall. Mice were first exposed to the testing chamber in a single 30 min habituation session, with the house light and white noise on. Magazine training began the following day. Reinforcer pellets were delivered into the internally illuminated magazine at the start of these sessions, and again every 30 s after each pellet was retrieved, until 50 pellets were delivered or 45 min passed, whichever occurred first. Mice remained in magazine training until they retrieved 50 pellets in a single session.

A three-stage aperture training followed. In the first stage, the food magazine was illuminated at the start of the session, and head entry resulted in pellet delivery, termination of magazine illumination, and illumination of the central nose poke aperture on the opposite wall. A response into the lit central nose poke terminated the nose poke illumination and led to illumination of the magazine and delivery of a pellet; a variable OR nose poke time of 0, 10, 20, or 40 centiseconds (cs) was required, randomized from trial to trial. Stages 2 and 3 followed the same general schedule, progressively increasing the OR duration array. In stage 2, the observing response array increased as a function of the rewards earned. When <15 reinforcers had been earned, an OR of 0, 10, 20, or 40 cs could be required. For reinforcers 16–25, OR times could be 0, 20, 30, or 50 cs. For the remainder of the session, OR durations could be 0, 20, 40, or 60 cs. Stage 3 OR durations were 20, 40, or 60 cs throughout the session. Transition from one stage to the next required mice to earn at least 30 reinforcers in a single session.

Mice began discrimination acquisition training the day following completion of stage 3. Here, an observing response in the central nose poke aperture (variable hold requirement of 20, 40, or 60 cs) initiated a trial, at which point the aperture holes flanking the center were both illuminated. Mice had 30 s to make an entry into one of those two apertures and were reinforced with two pellets for selecting the “correct” one and were punished with a 5 s time-out with all visual stimuli off for selecting the “incorrect” one. Which of the two apertures was selected to be correct was pseudorandomly assigned for each mouse and maintained throughout discrimination training. Failure to respond within the 30 s window was scored as an omission. Omissions were followed by a 5 s time-out. Correct and incorrect responses, as well as omissions, were followed by a 3 s intertrial interval (ITI), during which no apertures were illuminated. Each session lasted for 125 trials, 60 min, or until the discrimination criterion was met, whichever occurred first. The criterion for completing discrimination acquisition training was 80% correct responses in a sliding window of 20 trials. Mice that made fewer than five responses during 2 consecutive days of discrimination acquisition training were returned to stage 2 of aperture training, and then returned to the discrimination phase after passing aperture training again. Mice failing to respond in the reversal phase were not transferred back to training but were removed from the study. This did not apply to any mice tested in this experiment.

Reversal began the day after mice completed discrimination training. Testing conditions were identical, except that the aperture that resulted in pellet delivery during discrimination no longer produced reward and the opposite aperture now resulted in delivery of two pellets. Animals were tested until reaching the same performance criterion used during acquisition.

### Dependent variables

A series of calculated dependent variables is used to evaluate the performance of individual mice in the test; all variables are calculated separately for the acquisition and reversal stages. The number of trials required to reach the preset performance criterion is a key dependent variable, as this was the variable subject to the genome-wide linkage studies in the study by Laughlin et al. (2011) that led to the selection of *Syn3* as a positional candidate gene. Higher trait values are indicative of more difficulty with learning the initial or reversed rule.

To better understand the impact of contingency reversal on the number of trials required to reach performance criteria, a reversal fold-change variable was calculated as the number of trials to criterion in the reversal phase divided by the number of trials in the initial discrimination phase.

To investigate the influence of prior outcome on future choices, a behavioral flexibility score was calculated for each subject (Aarde et al., 2019). The behavioral flexibility score quantifies the trade-off between flexibility and stability in choice behavior. The correct response following a reward delivery is to make the same choice again (“win-stay” or success through stability), while the correct choice following a time-out is to make the opposite choice on the next trial (“lose-shift” or success through flexibility). We evaluated flexibility in response to positive and negative feedback by determining the relative likelihood of shifting behavior on the subsequent trial. Behavioral flexibility scores were calculated independently for positive and negative feedback as (shift trials – stay trials)/(shift trials + stay trials), and are bound by −1 and 1, with −1 indicating that the subject never shifted responding, and 1 indicating they always shift. In our task, the optimal flexibility score following positive feedback is −1; and optimal flexibility following negative feedback is +1.

Anticipatory responses are responses made to the correct or incorrect apertures after one trial has been completed but before the next one has been started by a satisfactory OR (i.e., during the ITI or trial initiation periods). These responses were counted and expressed as a fraction of the total number of trials initiated. As noted above, ORs of minimum duration were required to initiate a trial; in some cases, mice broke their OR before reaching the minimum criterion, and these OR failures were counted as a fraction of the number of presentations of each observing response duration.

A number of latency measures were collected to allow for deeper analysis of reversal learning performance and progression through stages of the task. Trial initiation latencies were measured as the time (in deciseconds, ds) from the start of a new trial to initiation of the observing response. Response latencies were measured as the time (in cs) between the presentation of the target apertures and the selection of one option; these were divided into correct and incorrect latencies, based on the outcome of the trial. Pellet retrieval latencies were measured as the time (in ds) between pellet delivery following a correct response and head entry into the magazine.

### Statistical analyses

Generalized estimating equations (GEE) were used to model the effect of genotype, sex, phase, and their interactions on performance. The model used the robust estimator for the covariance matrix, an unstructured correlation matrix, the maximum likelihood method of parameter estimation, type III model effects, and the χ^2^ Wald statistic for the full log quasi-likelihood function. Normality and linearity were assessed by Kolmogorov–Smirnov test and P-P (probability-probability) plots, respectively. In cases where normality and linearity were validated, a normal distribution with identity link function was used. In cases where linearity or normality were violated, transformations were conducted and gamma and inverse Gaussian distributions were tested. Intercept-only models (i.e., models lacking sex and genotype as predictors) were built for each variable to assess goodness of fit using the Corrected Quasi-Likelihood under Independence Model Criterion. Table 1 shows the relevant transformations and model information for each test. Phase was not included as a within-subject predictor in reversal fold change because this variable denotes a ratio between acquisition and reversal, so phase is inherently accounted for. Pairwise *post hoc* tests were used to evaluate significant model effects on all main and interaction effects, except the main effect of genotype; genotype was analyzed using a simple contrast *post hoc* analysis with homozygous wild-type mice as the reference group. All *post hoc* tests used the Sidak correction for multiple comparisons. Means in the text are estimated marginal means (EMMs) ± SEM unless otherwise stated. Data are presented in figures as raw ± SEM unless otherwise stated. Effect size was calculated for significant effects: ϕ (φ) was calculated when df* *=* *1 (interpretation: small = 0.10, medium = 0.30, large = 0.50) and Cramer’s *V* (*V*) was calculated when df  > 1 (small = 0.07, medium = 0.21, large = 0.35; Kim, 2017).

**Table 1:** GEE model parameters

Dependent variable	Transformation	Probability distribution	Link function	Within-subject effect	Prediction factor
Trials to criterion	None	Inverse Gaussian	Identity	Phase	Phase, genotype, sex
Reversal fold change	None	Inverse Gaussian	Identity		Genotype, sex
Anticipatory responses per trial	+1 (added to trials), log	Inverse Gaussian	Identity	Phase, side	Phase, side, genotype, sex
Omissions per trial	None	Normal	Identity	Phase	Phase, genotype, sex
Trial initiation latency	None	Inverse Gaussian	Identity	Phase	Phase, genotype, sex
Pellet retrieval latency	Box-Cox	Inverse Gaussian	Identity	Phase	Phase, genotype, sex
Response latency	Box-Cox	Inverse Gaussian	Identity	Phase, side	Phase, side, genotype, sex
Observing response failures per trial	+1 (added to observing response failures), log	Inverse Gaussian	Identity	Phase, observing response requirement	Phase, observing response requirement, genotype, sex
Flexibility score	+2 (added to score), log	Normal	Identity	Phase, feedback valence	Phase, feedback valence, genotype, sex

Estimation statistics (Ho et al., 2019) were used to further explore the data. Analyses were conducted using the DABEST package in R, and Cumming estimation plots were generated using the raw data, which were presented as individual dots in swarm plots on each chart. Vertical lines next to the swarm plots represent the mean ± SD for that group. Unpaired mean difference plots display the mean difference distributions ± SD on the *y*-axis, and the groups being compared on the *x*-axis.

## Results

### Trials to criterion

Mice required an average of 4.30 (SE = 0.486) sessions to complete the acquisition phase and 4.79 (SE = 0.388) to complete the reversal phase. There were no significant effects of genotype or sex, or any interaction between these variables.

As expected, mice required significantly larger numbers of trials to reach a performance criterion in the reversal versus acquisition stage (χ^2^_(1,_ *_N_*_=106)_ = 79.248, *p *<* *0.001, φ = 0.611; Table 2). Our model identified no significant main effect of genotype (χ^2^_(2,_ *_N_*_=106)_ = 4.918, *p *=* *0.086), or any significant genotype * phase interaction (χ^2^_(2,_ *_N_*_=106)_ = 4.957, *p *=* *0.086), though we did identify a significant genotype * sex interaction (χ^2^_(2,_ *_N_*_=106)_ = 5.997, *p *=* *0.050, *V* = 0.168). *Post hoc* analysis of EMMs revealed that homozygous-null males required significantly fewer trials to reach criterion performance compared with homozygous females (*post hoc* test, *p *=* *0.038) and heterozygous males (*post hoc* test, *p *=* *0.012). The difference between homozygous-null males (mean = 62.1, SE = 6.5) and females (mean = 93.1, SE = 7.90) is particularly interesting because it suggests that *Syn3* ablation may differentially affect males and females. There was no significant main effect of sex or any higher-level interactions involving sex (all χ^2^ < 4.957, all *p* > 0.05).

**Table 2:** Outcomes of GEE models on multiple task measures

	Trials to criteria			Reversal fold change			Anticipatory responses per trial			Omissions per trial			Trial initiation latency			Pellet retrieval latency			Response latency			Observing response failures per trial			Flexibility score		
	χ^2^	df	Sig.	χ^2^	df	Sig.	χ^2^	df	Sig.	χ^2^	df	Sig.	χ^2^	df	Sig.	χ^2^	df	Sig.	χ^2^	df	Sig.	χ^2^	df	Sig.	χ^2^	df	Sig.
Phase	79.248	1	<0.001*				102.584	1	<0.001*	34.308	1	<0.001*	1.258	1	0.262	0.238	1	0.625	224.982	1	<0.001*	13.536	1	<0.001*	2.848	1	0.092
Side							23.541	1	<0.001*										0.033	1	0.856						
Observing response requirement (ORR)																						754.522	2	<0.001*			
Feedback valence (FV)																									190.343	1	<0.001*
Genotype	4.918	2	0.086	7.958	2	0.019*	6.667	2	0.036*	0.14	2	0.932	3.663	2	0.16	1.439	2	0.487	0.56	2	0.756	6.086	2	0.048*	0.495	2	0.781
Sex	0.663	1	0.415	2.824	1	0.093	1.623	1	0.203	0.99	1	0.32	0.627	1	0.428	0.146	1	0.703	0.794	1	0.373	1.061	1	0.303	0.078	1	0.78
Phase * side							70.192	1	<0.001*										0.007	1	0.931						
Phase * ORR																						0.272	2	0.873			
Phase * FV																									162.468	1	<0.001*
Phase * genotype	4.957	2	0.084				0.146	2	0.93	2.467	2	0.291	0.502	2	0.778	0.003	2	0.998	7.254	2	0.027*	1.861	2	0.394	7.93	2	0.019*
Phase * sex	3.448	1	0.063				3.314	1	0.069	1.044	1	0.307	0.055	1	0.815	2.136	1	0.144	0.127	1	0.721	0.846	1	0.358	0.247	1	0.619
Side * genotype							4.037	2	0.133										0.143	2	0.931						
Side * sex							0.343	1	0.558										0.014	1	0.907						
ORR * genotype																						6.958	4	0.138			
ORR * sex																						0.59	2	0.745			
FV * genotype																									0.6	2	0.741
FV * sex																									9.039	1	0.003*
Genotype * sex	5.997	2	0.050*	0.995	2	0.608	1.592	2	0.451	0.07	2	0.966	0.423	2	0.81	2.096	2	0.351	2.608	2	0.271	5.408	2	0.067	0.211	2	0.9
Phase * side * genotype							2.282	2	0.32										0.086	2	0.958						
Phase * side * sex							1.872	1	0.171										0.991	1	0.319						
Phase * ORR * genotype																						2.244	4	0.691			
Phase * ORR * sex																						0.759	2	0.684			
Phase * FV * genotype																									3.084	2	0.214
Phase * FV * sex																									6.379	1	0.012*
Phase * genotype * sex	1.762	2	0.414				0.216	2	0.898	0.068	2	0.966	2.855	2	0.24	0.98	2	0.613	0.343	2	0.842	0.025	2	0.987	2.578	2	0.275
Side * genotype * sex							0.68	2	0.712										0.306	2	0.858						
ORR * genotype * sex																						1.671	4	0.796			
FV * genotype * sex																									4.396	2	0.111
Phase * side * genotype * sex							0.408	2	0.815										0.151	2	0.927						
Phase * ORR * genotype * sex																						3.223	4	0.521			
Phase * FV * genotype * sex																									0.161	2	0.922

**Figure 1.**
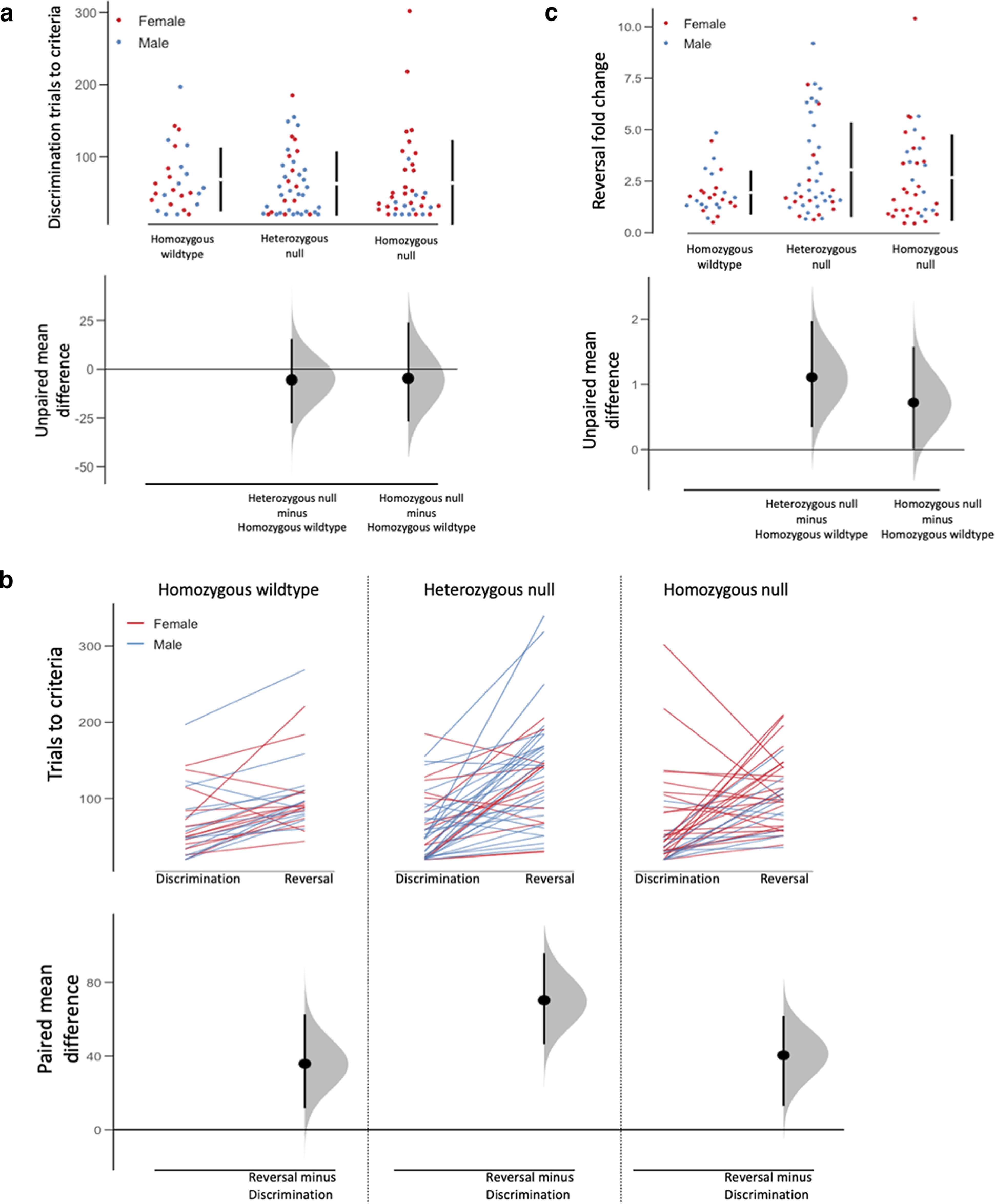
*Syn3* ablation negatively affects reversal learning performance. (***a***) Cumming estimation plot displaying discrimination performance between genotypes. The swarm plot in the top panel shows individual data points for each group with mean (±SD) displayed in the bar to the right. Unpaired mean differences comparing each mutant group to the wildtype control group are shown in the bottom panel. (***b***) Cumming estimation plot displaying discrimination and reversal performance within each genotype group. The line plot in the top panel shows individual data for mice in each genotype group. Paired mean differences comparing discrimination and reversal performance within each group are in the bottom panel. (***c***) Cumming estimation plot displaying reversal fold change score between genotype groups. The swarm plot in the top panel shows individual data points for each group with mean (±SD) displayed in the bar to the right. Unpaired mean differences comparing discrimination and reversal performance within each group.

An estimation statistics approach was used to further explore the data in light of our a priori hypothesis. All genotypes exhibited equivalent acquisition of the initial discrimination (Fig. 1*a*), and all experienced more difficulty with reaching performance criteria in the reversal, compared with the initial phase (Fig. 1*b*, bottom). However, the magnitude of increase in trials to criteria does not appear to be the same across genotypes. Specifically, heterozygous-null mice experienced considerable difficulty in meeting the criteria following contingency reversal.

To better visualize the reversal-specific impairments, a fold change statistic was calculated and visualized (Fig. 1*c*), and a GEE model was fit to the data. Genotype was found to significantly influence reversal fold change (χ^2^_(2,_ *_N_*_=106)_ = 7.958, *p *=* *0.019, *V* = 0.194; Table 2), and *post hoc* analysis revealed that reversal more substantially impaired performance in heterozygous mutant mice (mean = 2.89, SE = 0.37) compared with homozygous wild-type mice (mean = 1.95, SE = 0.26; *post hoc* test, *p *=* *0.037). The *post hoc* contrast comparing homozygous wild-type and homozygous mutant genotypes also trended on significance (*p *=* *0.064), and the Cumming estimation plot (Fig. 1*c*) shows only a very small portion of the mean difference distribution would support the null hypothesis that homozygous mutants are phenotypically equivalent to homozygous wild-type mice.

### Anticipatory responses

**Figure 2.**
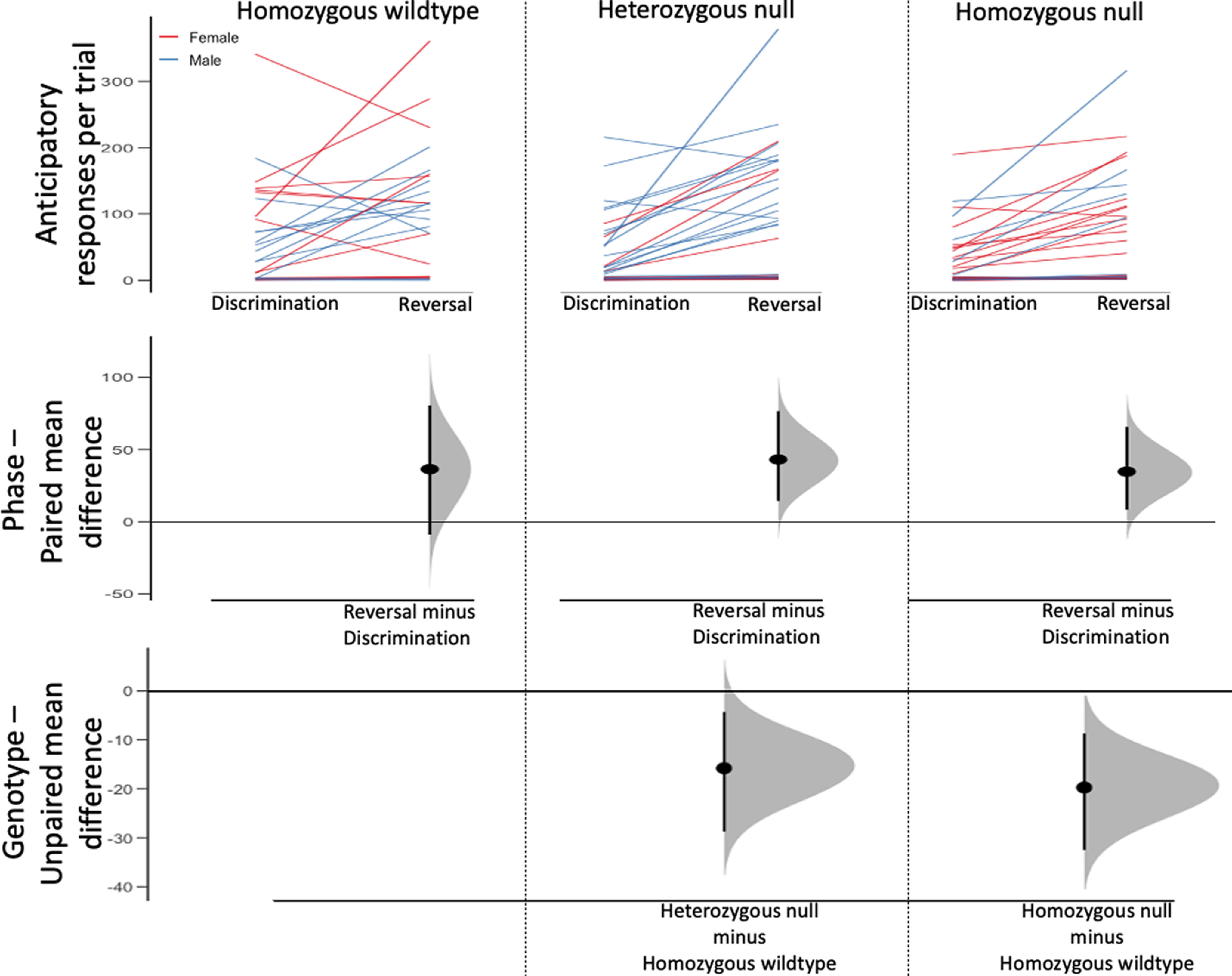
Contingency reversal increases anticipatory responses, but *Syn3* ablation reduces anticipatory responses compared to wildtype mice. Cumming estimation plot displaying anticipatory responses per trial in discrimination and reversal phases within each genotype group. The line plot in the top panel shows individual data for mice in each genotype group. Paired mean differences comparing discrimination and reversal performance within each group are in the middle panel. Unpaired mean differences comparing each mutant group to the wildtype control group are shown in the bottom panel.

**Figure 3.**
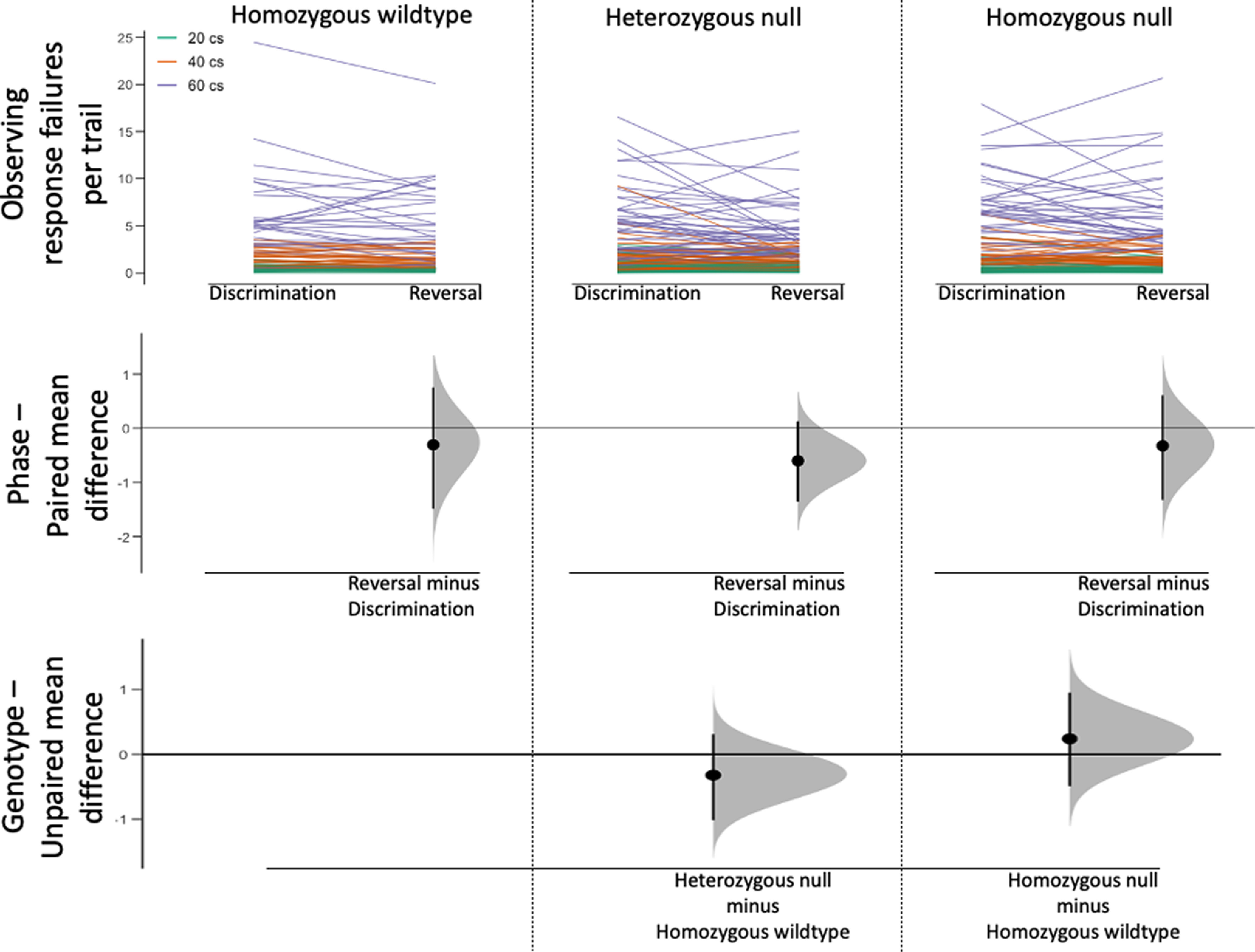
Observing response failures per trial show a probable difference between heterozygous null and homozygous null groups. Cumming estimation plot displaying observing response failures per trial in discrimination and reversal phases within each genotype group. The line plot in the top panel shows individual data for mice each genotype group. Paired mean differences comparing discrimination and reversal performance within each group are in the middle panel. Unpaired mean differences comparing each mutant group to the wildtype control group are shown in the bottom panel.

For the results of model effects for anticipatory responses per trial, see Figure 3. There were generally more anticipatory responses made during the reversal phase (main effect of phase: χ^2^_(1,_ *_N_*_=106)_ = 102.584, *p *=* *0.000, φ = 0.491; Table 2). In addition, more anticipatory responses were made on the side that was rewarded during discrimination, regardless of current phase (χ^2^_(1,_ *_N_*_=106)_ = 23.541, *p *<* *0.001, φ = 0.236). We did not detect a main effect of sex or any higher-level interactions involving sex (all χ^2^ < 4.037, all *p* > 0.05; Table 2). There was a main effect of genotype for anticipatory responses (χ^2^_(2,_ *_N_*_=106)_ = 6.667, *p *=* *0.036, *V* = 0.089; Fig. 2); however, the direction of the effect was that *Syn3* deletion reduced premature responding. Homozygous wild-type mice made more anticipatory responses than the heterozygous mice (*post hoc* test, *p *=* *0.024), and although the Cumming estimation plot comparing genotypes (Fig. 2, bottom) suggests a mean difference separation between the homozygous-null and homozygous wild-type groups, our *post hoc* analysis evaluating this difference in our model did not reach to the level of significance (*p *=* *0.098). We believe that this is a consequence of adjustments made to the EMM used for *post hoc* analyses within the model. EMMs are adjusted to correct for the influence of other factors in the model to specifically evaluate the variance explained by the factor being tested.

To further investigate this issue, nested mean tables were generated to visualize the pattern of descriptive means as a function of each factor level. Descriptive means at the sex * genotype level showed the same rank-order patterns identified in our *post hoc* and estimation analyses. Specifically, females displayed the same pattern of EMMs (homozygous wild type > homozygous null > heterozygous null), while males matched the pattern seen in genotype-based descriptive means (homozygous wild type > heterozygous null > homozygous null). We next examined genotype * sex EMMs and found the same sex * genotype pattern. EMMs for genotype were then generated excluding sex from the model, and the pattern was found to match that of the descriptive means, suggesting that the inclusion of sex in the model accounted for the observed effects.

### Omissions

We found a main effect of phase (χ^2^_(1,_ *_N_*_=106)_ = 34.308, *p *=* *0.000, φ = 0.402; Table 2); mice omitted fewer trials in reversal than in discrimination. No other differences were observed for trial omissions.

### OR failures

There was a main effect of phase, such that more observing response failures occurred during the acquisition stage, than during reversal (χ^2^_(1,_ *_N_*_=106)_ = 13.536, *p *< 0.001, φ = 0.146; Table 2). Longer observing response requirements resulted in more failures per trial (χ^2^_(2,_ *_N_*_=106)_ = 754.522, *p *<* *0.001, *V* = 0.770; Fig. 3, top), and *post hoc* assessment revealed significant differences among all hold requirements (all pairwise *p *<* *0.001). Genotype was also found to significantly impact observing response failures (χ^2^_(2,_ *_N_*_=106)_ = 6.086, *p *=* *0.048, *V* = 0.069); however, *post hoc* analyses comparing each mutant type to the wild-type control did not reveal pairwise differences. The Cumming estimation plot comparing genotypes (Fig. 3, bottom) shows that heterozygous-null and homozygous-null groups deviate from the homozygous wild-type group in opposite directions, suggesting that the main effect of genotype identified in the GEE model is a difference between heterozygous-null and homozygous-null groups.

### Latency measures

Latency model effects are reported in Table 2. Neither trial initiation latency nor pellet retrieval latency was found to vary as a function of phase, genotype, sex, or their interactions (all *p *>* *0.05).

Response latencies were longer in acquisition than in reversal (χ^2^_(1,_ *_N_*_=106)_ = 224.982, *p *<* *0.001, φ = 0.729). There was also a significant phase * genotype interaction (χ^2^_(2,_ *_N_*_=106)_ = 7.254, *p *=* *0.027, *V* = 0.093). All mice reduced their response latencies between the acquisition and reversal phases, but the effect was most pronounced in the heterozygous group. There were no differences in latency as a function of correct or incorrect responses (no effects of side, all *p *>* *0.05; Table 2).

### Behavioral flexibility score

**Figure 4.**
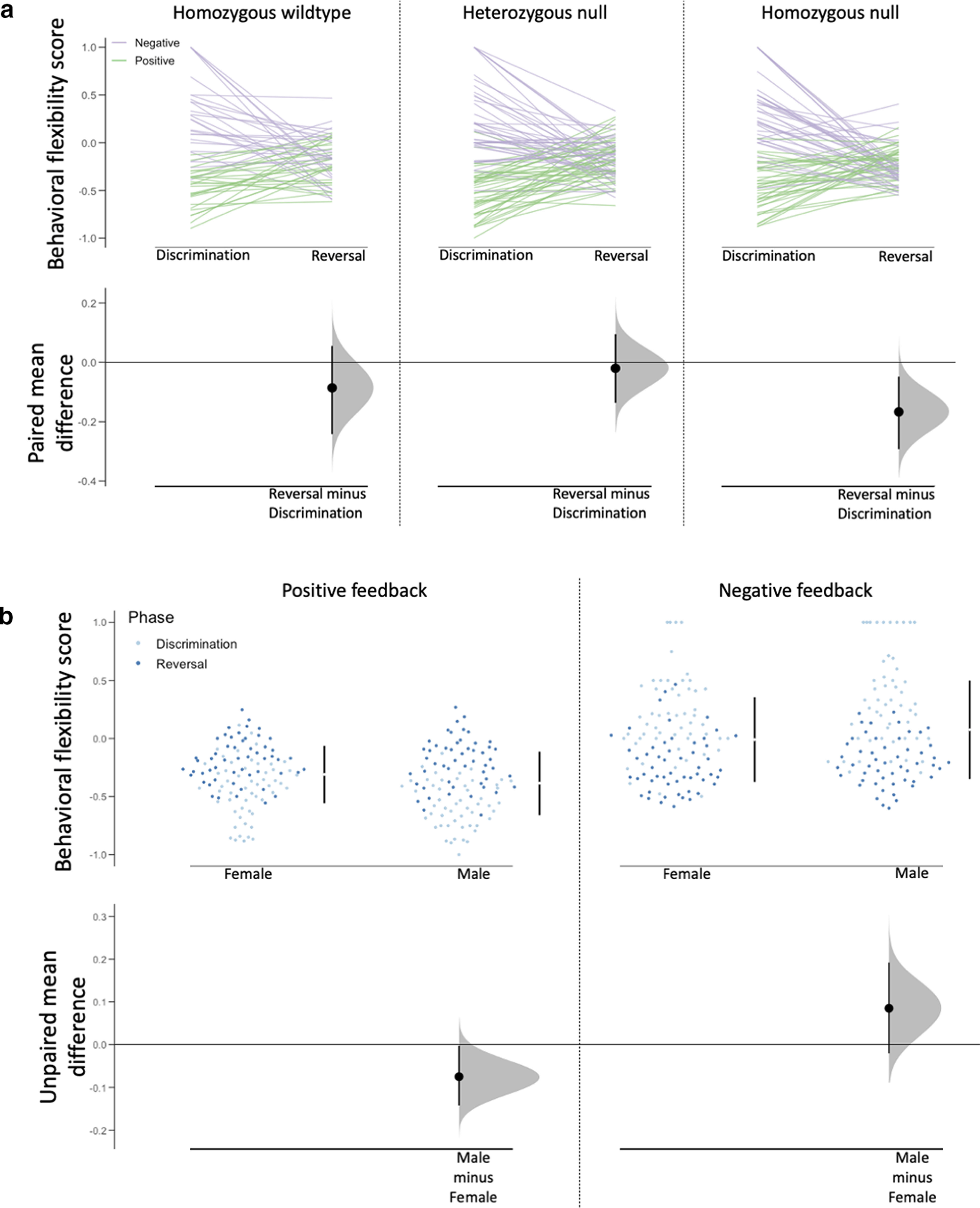
*Syn3* ablation reduces flexible behavior during contingency reversal. (***a***) Cumming estimation plot displaying behavioral flexibility during discrimination and reversal within each genotype group. The line plot in the top panel shows individual data points for mice in each genotype group. Paired mean differences comparing discrimination and reversal performance within each group. ***b*** Cumming estimation plot displaying behavioral flexibility discrimination and reversal in both sexes. The swarm plot in the top panel shows individual data points for each group with mean (±SD) displayed in the bar to the right. Unpaired mean differences comparing discrimination and reversal performance within each group.

Contingency reversal impacted feedback integration in a valence-dependent manner (phase * feedback valence interaction: χ^2^_(1,_ *_N_*_=106)_ = 162.468, *p *<* *0.001, φ = 0.875; Table 2). In discrimination acquisition, mice exhibited stable behavior following positive feedback (mean = −0.50, SE = 0.023) and flexible behavior following negative feedback (mean = 0.28, SE = 0.037), but this separation was largely lost in the reversal phase (mean positive = −0.20, SE = 0.021; mean negative = −0.19, SE = 0.023). A phase * genotype interaction was identified (χ^2^_(2,_ *_N_*_=106)_ = 7.930, *p *=* *0.019, *V* = 0.137), and pairwise *post hoc* analysis revealed a more negative flexibility score for null mice in reversal compared with their flexibility in discrimination (*p *=* *0.034; Fig. 4*a*), suggesting the behavior of *Syn3*-null mice were less flexible in reversal than in acquisition. We also identified feedback valence * sex (χ^2^_(1,_ *_N_*_=106)_ = 9.039, *p *=* *0.003, φ = 0.206) and phase * feedback valence * sex (χ^2^_(1,_ *_N_*_=106)_ = 6.379, *p *=* *0.012, φ = 0.173) interaction effects. Male mice were less likely to change responses after positive feedback and were more likely to update behavior after negative feedback compared with females (i.e., less flexibility after positive feedback and more flexibility after negative feedback; Fig. 4*b*), but as with the phase * feedback interaction, this difference only occurred in the discrimination acquisition phase, and differences were not apparent between groups in reversal.

## Discussion

In this experiment, we tested the influence of the *Syn3* gene on reversal learning performance in C57BL/6N mice using a global knockout strategy; based on previous systems genetics analysis of this trait in the BXD population (Laughlin et al., 2011), we hypothesized that mice lacking *Syn3* would display deficits in reversal learning performance compared with wild-type mice. Portions of our data support that hypothesis, but the precise effect of synapsin III deletion on reversal learning is nuanced and worthy of further exploration.

We found some evidence that reversal learning ability varies as a function of genotype. When a fold-change variable that isolates reversal phase-specific aspects of performance was calculated, significant differences were apparent between the genotypes. Specifically, mice carrying one or two null alleles experienced a larger proportional increase in trials to criterion in reversal compared with wild-type mice. Furthermore, *Syn3*-null mice exhibited less flexible behavior in reversal compared with the initial discrimination (as shown by the flexibility score analysis), again suggesting a reversal-specific deficit. These findings are consistent with previous observations in BXD mice; Laughlin et al. (2011) found that the trials to criteria under reversal testing was associated with the chromosome 10 QTL. When that measure was regressed on trials to criteria for the acquisition phase at the individual-subject level, and the mean residual scores per strain (the portion of variance in the reversal data that could not be accounted for by variance in the acquisition data) were submitted to a second genome scan, they found statistically identical results, showing that the association was specific to some reversal-specific feature of behavior.

The rate of learning under the reversal condition was not the only phenotype affected by *Syn3* deletion. Wild-type mice made more anticipatory responses than either mutant group. This measure is conceptually similar to the premature responding phenotype measured in the five-choice serial reaction time task (5CSRTT), often interpreted as an indicator of waiting impulsivity (Dalley and Ersche, 2019). At a minimum, the dissociation between behavioral flexibility assessed in reversal (impaired) and premature responding (reduced) in *Syn3*-null mice suggests that these two traits are influenced by separate genetic architectures, as existing evidence already suggested (Nautiyal et al., 2017). That hypothesis is further supported by recent observations that measures of behavioral flexibility and premature responding are not genetically correlated in the collaborative cross-recombinant inbred panel and their inbred founder strains (Bailey et al., 2021). Moreover, *Syn3* negatively affected the ability to maintain the variable duration OR. How, and/or whether, this phenotype is related to their reduced premature responding is unclear. It is also worth noting that premature responding in the 5CSRTT is typically penalized by a time-out period that lengthens the time before the start of the next trial, so the inhibition of premature responses is required for optimal task performance. That is not the case in our reversal learning task; premature responses are recorded but have no programmed consequences.

In our procedure, premature responses could occur during the intertrial interval (before the OR becomes available) or during the trial initiation period (after the OR is available but before the OR duration criterion is met). Responses occurring during the trial initiation period could signal a failure to attend to the discriminative stimulus indicating a reward is now available for the correct choice (i.e., the OR aperture light turns off and the flanking lights turn on). Responding during the intertrial interval is somewhat more difficult to interpret, partially because we are unsure whether these responses occurred following an attempted OR or are independent of an OR. Observing response failures, on the other hand, occurred at very low levels when the OR requirement was low, indicating that the average response duration was likely higher than the minimum OR requirement. As the duration criteria increase, however, failures occur more often. Perfect success could be generated by sustaining the aperture response until the aperture light turns off on all trials, but clearly this is not the strategy used by the mice. Instead, they sustain the OR for some variable period of time that produces almost certain success at the shortest OR duration, and much lower success rates with more sustained response requirements.

Elaborating the microstructure of premature response patterns in reversal learning would lend clarity to the relation between the observing response and the premature or anticipatory response in preparations like ours that require a sequence of behavioral responses to receive a reward. If an OR failure is followed by another OR attempt, the subject is likely still attending to the relevant aperture. If an OR failure is followed by one or more anticipatory responses, the subject has shifted behaviors without attending to the lack of shift in discriminative stimuli. Though both patterns could be classified inefficient, they denote different underlying behavioral strategies. The ability to sustain a response for a sufficient duration measures a different dimension of inhibitory control than the ability to wait for choice conditions to be met before making a response. It is possible that the variable OR duration presented a greater challenge for *Syn3*-null mice by introducing uncertainty into the behavioral response itself. This is functionally distinct from responding in the choice apertures before a choice is available, in which *Syn3* ablation seems to confer an advantage.

### Role for DA

Given the known regulatory role of *Syn3* on DA transmission (Kile et al., 2010), we can cautiously link our observed results to altered DA dynamics. Laughlin et al. (2011) found that *Syn3* expression in the neocortex, hippocampus, and striatum was correlated with reversal learning performance (low Syn3 expression was associated with poor reversal learning performance). Synapsin III negatively regulates DA release by controlling the transfer of synaptic vesicles from the reserve pool to the ready-releasable pool (Feng et al., 2002), and mice lacking *Syn3* show enhanced striatal phasic DA release compared with wild types (Kile et al., 2010). Presumably, *Syn3* deficiency would also induce enhanced release in hippocampus and neocortex, but these effects, to our knowledge, have not been explicitly tested. Inducible and region-specific knockout strategies could be leveraged to better understand the relative contributions of corticolimbic and corticostriatal circuits to reversal learning.

Dopaminergic tone has been implicated in the ability to reverse a learned discrimination (Klanker et al., 2013). Klanker et al. (2015) identified a key role for ventromedial striatal DA responses to positive feedback while animals acquired a spatial reversal task. In rats that eventually acquired the reversal rule, the first rewarded reversal trial induced a spike in DA (measured via fast-scan cyclic voltammetry) concurrent with reward delivery; in the subsequent trial (i.e., the first trial in which positive feedback can be used to update behavior), an increase in cue-evoked DA was observed. In rats that did not acquire reversal, the first reward induced the same DA spike, but DA release did not shift to cue presentation in the next trial. This effect was specific to positive feedback; DA release during an incorrect trial (i.e., one followed by no reward delivery) or the following trial did not differ between rats that acquired the reversal and those that did not. A change point was defined as the trial at which the cumulative record maximally deviated from a line drawn from the origin to the end of the record (Klanker et al., 2015). No differences were observed in cue-evoked DA release in trials before and after the change point, but reward-evoked DA release decreased in trials following the change point. Positive feedback increased cue-evoked DA release on the subsequent trial only before the change point, suggesting feedback-induced shifts in intratrial timing normalize across the learning curve to a level where cue-evoked DA stabilizes, and the learned behavior is reliably expressed. Dopaminergic tone is dynamically engaged throughout the learning curve, and small shifts in the timing of release can aid in reorganization of previously learned behavior in response to positive feedback.

Yawata et al. (2012) demonstrated independent roles for the direct (D_1_ receptor-expressing) and indirect (D_2_ receptor-expressing) dopamine-mediated pathways of the basal ganglia by reversibly inducing pathway-specific blockade of neurotransmission in the nucleus accumbens using doxycycline in transgenic mice. Interference with the D_1_ receptor-expressing direct pathway impaired acquisition of a novel visual discrimination, though no impairment in acquisition was observed when the D_2_ receptor-expressing indirect pathway was blocked. Blocking neurotransmission in either the direct or indirect pathway interfered with reversal of the previously learned discrimination, but only inhibiting the indirect pathway neurons increased perseverative errors. This pattern of findings was recapitulated when the direct or indirect pathways were unilaterally blocked via doxycycline, then the contralateral side was treated with D_1_ or D_2_ agonists or antagonists. Antagonism of accumbal D_1_ receptors interfered with the acquisition of initial and reversed discriminations but did not alter perseverative errors, while D_2_ agonism selectively hindered reversal performance by increasing perseverative errors (Yawata et al., 2012). Together, these results suggest that the D_1_-mediated direct pathway in the basal ganglia is involved in the acquisition of operant responses more generally, while DA release onto the D_2_-mediated indirect pathway is specifically implicated in behavioral flexibility.

Lee et al. (2007) demonstrated that in monkeys systemic antagonism of D_2_/D_3_, but not D_1_/D_5_, specifically impaired performance following reversal of a previous learned visual discrimination without altering the ability to acquire novel discriminations. In rats, intra-accumbal D_1_, but not D_2_, antagonism disrupted set shifting (a measure of cognitive flexibility), while D_2_, but not D_1_, agonism impaired performance on a reversal task without disrupting initial discrimination (Haluk and Floresco, 2009). Although one might expect agonism and antagonism of D_2_ receptors to have opposite effects, conceptual and experimental evidence points to a convergent role of the change in dopamine activity to drive behavioral effects. Because D_2_ receptors are found both presynaptically and postsynaptically, agonism will inhibit further dopamine release via autoreceptors, while antagonism will interfere with postsynaptic activation even as presynaptic release is disinhibited. In both cases, phasic dopamine activity is disrupted, and reversal learning is impaired.

Phasic DA release has been proposed to serve as a teaching signal, encoding a prediction error signal whereby unexpected outcomes generate an increase in phasic DA (Schultz, 2019). Steinberg et al. (2013) demonstrated that optogenetic stimulation of VTA DA neurons concurrent with reward delivery produced long-lasting enhancement of cue-induced reward seeking. Saunders et al. (2018) replicated and extended these findings, demonstrating that VTA, but not SNc, DA release paired with cue presentation evoked conditioned stimulus approach behavior. Further, pairing optogenetic DA stimulation with reward in a behavioral economic procedure shifts the demand curve rightward and upward, indicating higher subjective value to the reward and enhanced motivation to obtain it (Schelp et al., 2017). Synapsin III negatively regulates dopamine release by regulating the transfer of synaptic vesicles from the reserve pool to the ready-releasable pool (Feng et al., 2002), and its absence enhances phasic dopaminergic tone (Kile et al., 2010). Thus, mice carrying one or more null *Syn3* alleles may experience the initial acquisition of a discrimination differently, and our test may simply not be sensitive enough to detect any reinforcement learning phenotypes during the initial acquisition stage.

### Relevance to substance use disorder

Stimulant drugs of abuse are widely known to act by enhancing dopaminergic activity, either by blocking DA transport (DAT; e.g., cocaine) or by promoting the reverse transport of DA (e.g., amphetamine and methamphetamine). A recent systematic review and meta-analysis of the literature regarding dopaminergic alterations in stimulant users found several noteworthy changes in DA dynamics, including reduced overall DA release, reduced DAT availability, reduced D_2_/D_3_ receptor availability, and possibly reduced DA synthesis (Ashok et al., 2017). Notably, striatal D_2_/D_3_ receptor availability has been negatively correlated with reversal learning performance (Dalley et al., 2007; Groman et al., 2011; Laughlin et al., 2011). Theoretically, *Syn3* deletion, by amplifying phasic DA release, may lead to compensatory decreases in D_2_-like receptors in forebrain regions, thereby conferring a less flexible phenotype.

### Sex differences in learning

Our analysis identified several sex-related effects in performance. Specifically, we found that, during initial acquisition of the discrimination, homozygous-null male mice required fewer trials to reach criterion than did females of the same genotype. In addition, we found that males engaged in more optimally flexible behavior during discrimination acquisition learning (i.e., males more often made the same choice after positive feedback and switched choices after negative feedback more often than did females), but both sexes responded equivalently to both types of feedback during contingency reversal. This suggests the importance of sex differences in learning strategies during the initial discrimination phase.

Previous research examining sex differences in learning strategy is mixed. Using a probabilistic reversal learning task, Harris et al. (2021) found that male rats were more sensitive than females to negative feedback throughout training, with no difference in sensitivity to positive feedback or overall learning rate. Chen et al. (2021) tested mice in a visual bandit task and found that females learned faster than males and engaged in a stable strategy throughout the task, while males were more sensitive to previous outcomes and changed their strategy over time. Notably, there were no sex differences in visual bandit performance when reward probabilities were deterministic (Chen et al., 2021), which most closely reflects the testing conditions used here. A study using the rodent version of the Iowa Gambling Task found that males learned at a faster rate than females (van den Bos et al., 2012), but a modified version of the task used by Peak et al. (2015) found that females optimized choice behavior faster (Orsini and Setlow, 2017). Despite difficulty in making an overarching statement about sex differences in learning, it is clear from this pattern of results that males and females use task-relevant information differently, resulting in task-specific sex differences in strategy optimization.

### Limitations

Our ability to scale our findings up to findings in humans is limited, in part, by the simplicity of our task. In the real world, humans are often required to make decisions based on uncertain information and update behavior as a function of feedback in real time. We use a deterministic task, while tests used in humans often incorporate probabilistic reward delivery to introduce uncertainty and find differential results as a function of that uncertainty (Chen et al., 2021). Likewise, intersession (overnight) consolidation can influence learning (Varga et al., 2014; Alizadeh Asfestani et al., 2018), and our task used a multisession, between-sessions training and testing strategy. Future research attempting to link our mouse genetic findings with human research may need to address these issues.

### Conclusions

Here we have demonstrated a role for *Syn3*, encoding synapsin III, in behavioral flexibility. C67BL/6N mice lacking functional *Syn3* alleles experienced a greater proportional cost of contingency reversal than wild-type mice and engaged in less flexible responding during the reversal phase but made fewer anticipatory responses; however, it is important to note that the reported pattern of effects may well be dependent on the genetic background studied (Sittig et al., 2016). This suggests that *Syn3* homozygous-null mice were less adaptable to changes in contingency, but this effect was independent of their waiting impulsivity phenotype.

## Synthesis

Reviewing Editor: Christie Fowler, University of California, Irvine Decisions are customarily a result of the Reviewing Editor and the peer reviewers coming together and discussing their recommendations until a consensus is reached. When revisions are invited, a fact-based synthesis statement explaining their decision and outlining what is needed to prepare a revision will be listed below. The following reviewer(s) agreed to reveal their identity: NONE.

This manuscript has been reviewed by two experts in the field. Both reviewers felt the data presented are a significant advance and of interest to the field. During the consultation session, the reviewers concurred that additional revisions are necessary to properly evaluate the experimental findings. Specific comments are as follows:

Reviewer 1

The authors sought to determine whether synapsin III (syn3) contributes to reversal learning performance in a contingent manner after evidence for positive correlation between the trait of this gene, and its expression, and reversal learning performance across mouse strains, and given the importance of deficits in this behavior in people with psychiatric conditions. The authors tested the impact of reduced Syn3 expression in performing a reversal learning task, where mice had 2, 1, or 0 copies of the allele, in both males and females. This strategy is excellent since not only the impact of loss of function of the gene, which could impact development, but reduced expression (more likely seen in humans than total loss), is tested. Furthermore, the statistical approach regarding flexibility measures are important, and well-documented. That a reversal effect was seen on total trials to criterion is an important first-step in the analysis, confirming the sensitivity of the task to reversal effects. They observed a trend effect of genotype and phase* genotype interaction (p=0.086), and an interaction between genotype and sex. They observed that Syn3 mice with 1 expression took more trials to learn the reversal task, than mice with 2 copies of the gene, while limited effect was seen with 0 copies.

Major Comments:

The authors provided only 50 trials per session during initial training, but 125 during testing in the task - did mice consume 125 pellets in a single session? More than 125 trials were needed for initial learning and reversal for many of the mice. How many sessions were required, since duration and overnight consolidation could impact learning.

The authors raise the importance of behavioral flexibility in terms of human diseases, but when humans are tested in such reversal learning tasks, they typically occur using probabilistic learning feedback so 1) the task is not too easy, 2) the impact of misleading information can be assessed. They also test in a single session vs. over multiple sessions the latter which include overnight consolidation. What impact do the authors think such misleading information, or overnight consolidation processes might have impacted their findings, and particular with relevance to human conditions?

Why were mice ‘offered’ 0.5 g of reinforcers in their homecage prior to experiments? Did they eat it? Was their eating of it differ by genotype that might of affected performance?

Given the genotype * sex interactions in learning observed, it is surprising the authors do not provide information on whether 1 genotype of each sex exhibited particular differences in learning - especially as females with 0 copies at the initial learning, and such analyses were conducted for anticipatory responding.

It is laudable that the authors test for sex effects and interactions between genotype, and indeed they see several effects and interactions. These are not discussed anywhere however, which is a missed opportunity that should be addressed, especially given sex-differences described by others in learning strategies (PMID 33125868; 30143781).

It is appreciated that the authors obtained these mice from Jackson Labs, but would be useful to cite confirmation of differing Syn3 levels, in addition to differing dopamine or vesicle pool levels in the manuscript to support their assertions.

Minor comments

Syn3 should be spelled out on first use in the introduction as in the abstract.

Likewise with DA (especially as referred to as dopamine sometimes), and GABA, VMAT2, etc.

Given the interest in dopamine and reversal learning, it is surprising the authors do not cite the work of Jentsch and colleagues in the introduction, particularly PMIDs 29527566, 21593313.

Please provide numbers of males and females by genotype, rather than altogether.

Given the authors are describing differences between groups of reversal minus initial learning, effect sizes would be useful to the reader to affirm such changes.

Reviewer 2

In this manuscript the authors want to correlate behavioral flexibility with the expression of the gene encoding Synapsin III, a vesicular protein which regulates phasic dopamine release.

To this aim they compared learning performances (both during acquisition and reversal) in three different mice genotypes, Wild Type, Syn3 heterozygous and Syn3 null mice.

The authors conclude that Syn 3 expression negatively affected behavioral flexibility in a reversal specific manner but may have reduced waiting impulsivity.

The paper is well written, the experiments well organized and described and the results are sound.

I have only to minor comments:

1. On page 10 (materials and methods section) the description of the reversal fold-change variable should be explained more clearly. It is not intuitive how a negative value could be generated from a ratio (number of trials to criterion in the reverse phase divided by the number of trials in the initial discrimination phase).

2. Page 22, first line after paragraph title ’Relevance to substance use disorder’: stimulant not simulant

## Author Response

We thank the reviewers for their thorough and constructive comments, which have elicited changes that we feel improve the impact of the manuscript. Below, we summarize the comments of each reviewer, the changes we made to the manuscript and the location of those changes.

Reviewer 1

Major Comments:

1. The authors provided only 50 trials per session during initial training, but 125 during testing in the task - did mice consume 125 pellets in a single session? More than 125 trials were needed for initial learning and reversal for many of the mice. How many sessions were required, since duration and overnight consolidation could impact learning.

Author response: On no occasion did mice consume 125 pellets in a single session. This would have required a subject to correctly perform all trials. Given that the criteria for passing either the discrimination or reversal phases was set to 80% correct in a sliding window of 20 trials a mouse performing perfectly would have met the criteria after 20 trials, and the session would have ended. Information on the total number of sessions required for both testing phases has been added to the results section (lines 233-235).

2. The authors raise the importance of behavioral flexibility in terms of human diseases, but when humans are tested in such reversal learning tasks, they typically occur using probabilistic learning feedback so 1) the task is not too easy, 2) the impact of misleading information can be assessed. They also test in a single session vs. over multiple sessions the latter which include overnight consolidation.

Author response: Use of a probabilistic reversal task would increase the complexity of the task and allow for a more nuanced examination of decision making under uncertain conditions that more closely resemble those studied in humans. The parameters of the reversal task we used were selected to match those reported in the study that identified Syn3 as a candidate gene (Laughlin et al., 2011). Our rationale for selecting the reversal task has been made clearer (lines 120-121).

3. What impact do the authors think such misleading information, or overnight consolidation processes might have impacted their findings, and particular with relevance to human conditions?

Author response: A limitations section has been added to the Discussion to address these issues (lines 492-500).

4. Why were mice ‘offered’ 0.5 g of reinforcers in their homecage prior to experiments? Did they eat it?

Author response: Mice were offered reward pellets in their home cages prior to experiments so that the pellets were not novel when first delivered in the chamber. Some mice have a tendency not to approach novel objects or foods and we did not want this to interfere with operant training. All mice consumed the pellets in the home cage. This information has been added (lines 126-127).

5. Was their eating of it differ by genotype that might of affected performance?

Author response: Mice did not differ in their home cage consumption of pellets, nor did we observe pellets remaining in the food magazine within the chamber. We conclude that mice find the pellet rewards palatable, regardless of genotype. This information has been added (line 127).

6. Given the genotype * sex interactions in learning observed, it is surprising the authors do not provide information on whether 1 genotype of each sex exhibited particular differences in learning - especially as females with 0 copies at the initial learning, and such analyses were conducted for anticipatory responding.

Author response: A more detailed analysis of the genotype*sex interaction on trials to criterion has been added (lines 240-245).

7. It is laudable that the authors test for sex effects and interactions between genotype, and indeed they see several effects and interactions. These are not discussed anywhere however, which is a missed opportunity that should be addressed, especially given sex-differences described by others in learning strategies (PMID 33125868; 30143781).

Author response: A section discussing sex differences in learning has been added to the Discussion (lines 469-491).

8. It is appreciated that the authors obtained these mice from Jackson Labs, but would be useful to cite confirmation (or provide direct evidence) of differing Syn3 levels, in addition to differing dopamine or vesicle pool levels in the manuscript to support their assertions.

Author response: This Syn3 strain was developed and phenotyped by the Knockout Mouse Project. It harbors a reporter-tagged deletion alleles. Details have been added (lines 99-102).

Minor comments

1. Syn3 should be spelled out on first use in the introduction as in the abstract.

Author response: This change has been made (lines 45-46).

2. Likewise with DA (especially as referred to as dopamine sometimes), and GABA, VMAT2, etc.

Author response: This change has been made (lines 62-64).

3. Given the interest in dopamine and reversal learning, it is surprising the authors do not cite the work of Jentsch and colleagues in the introduction, particularly PMIDs 29527566, 21593313.

Author response: These citations have been added (lines 77-82).

4. Please provide numbers of males and females by genotype, rather than altogether.

Author response: This change has been made (lines 114-115).

5. Given the authors are describing differences between groups of reversal minus initial learning, effect sizes would be useful to the reader to affirm such changes.

Author response: Effect sizes were calculated for significant effects and are reported in text with parenthetical statistics. A description of how the effect sizes were obtained has been added to the methods section (lines 222-224).

Reviewer 2

1. On page 10 (materials and methods section) the description of the reversal fold-change variable should be explained more clearly. It is not intuitive how a negative value could be generated from a ratio (number of trials to criterion in the reverse phase divided by the number of trials in the initial discrimination phase).

Author response: Sentences referring to traditional difference scores and negative values were removed for clarity. A negative value is not possible in our calculation of reversal fold-change, which was selected over a traditional difference score for that reason (between lines 179-180).

2. Page 22, first line after paragraph title ‘Relevance to substance use disorder’: stimulant not simulant

Author response: This change has been made (line 459).
